# Clinical significance of PD-L1 protein expression on tumor-associated macrophages in lung cancer

**DOI:** 10.1186/2051-1426-3-S2-P415

**Published:** 2015-11-04

**Authors:** Kurt Alex Schalper, Daniel Carvajal-Hausdorf, Joseph McLaughlin, Vamsidhar Velcheti, Lieping Chen, Miguel Sanmamed, Roy S Herbst, David L Rimm

**Affiliations:** 1 Yale University, New Haven, CT, USA; 2 Cleveland Clinic, Cleveland, OH, USA; 3 Yale Cancer Center, Yale School of Medicine, Yale University, New Haven, CT, USA

## Introduction

Tumor PD-L1 expression is associated with increased tumor-infiltrating lymphocytes (TILs) in diverse solid tumors, including lung cancer. In addition, PD-L1 upregulation in tumor and/or stromal immune cells has been associated with increased clinical benefit to PD-1/PD-L1 axis blockers. The significance of PD-L1 expression in different immune cell subpopulations at the tumor microenvironment remains poorly understood. Here, we measured PD-L1 protein specifically in tumor-associated macrophages using objective methods and analyzed its clinical significance in human lung cancer.

## Methods

Using multiplexed quantitative immunofluorescence (QIF) we measured the levels of PD-L1 protein (clone-E1L3N, Cell Signaling technology) in 554 stages I-IV FFPE lung carcinomas represented in two tissue-microarrays, one from Yale University [YTMA79, n=204] and one from Greece [YTMA140, n=350]. PD-L1 was measured using multispectral QIF in CD68-positive cells (macrophages, clone-KP1, DAKO) or in tumor cells (clone-AE1/AE3, DAKO) based on fluorescence co-localization. The levels of CD3 (T cells, clone-E272, Novus), CD8 (cytotoxic T cells, clone-C8/144B, DAKO) and CD20 (B-lymphoctes, clone-L26, DAKO) were also determined in the tumor samples. Associations between the macrophage and tumor PD-L1 levels, clinico-pathological variables and survival were analyzed.

## Results

In lung cancer samples, PD-L1 was detected in both macrophages and tumor cells with a predominant membranous staining pattern. Overall, the levels of PD-L1 in the CD68+ population were lower than in tumor cells in both cohorts (834 ± 314 AU vs 1113 ± 427 AU, in the Yale cohort, P < 0.001; and 895 ± 451 AU vs 1069 ± 542 AU in the Greek set, P < 0.001). In both collections, the macrophage and tumor PD-L1 signal showed a positive non-linear association (Linear regression coefficient [R^2^]=0.311 in the Yale cohort and R^2^=0.43 in the Greek collection). Using the median score as cutpoint, elevated macrophage PD-L1 content was significantly associated with high CD8+ and CD20+ cells in both cohorts. In addition, increased macrophage PD-L1 signal was marginally associated with longer overall survival in the Yale set (HR=0.676 [CI:0.450-1.081], P=0.05), but not in the Greek group (HR=0.790 [CI:0.560-1.109], P=0.17).

## Conclusion

PD-L1 is expressed in tumor-associated macrophages of human lung carcinomas. The macrophage PD-L1 signal is lower than in tumor epithelial cells and they show a positive association. Elevated macrophage PD-L1 is prominently associated with increased cytotoxic T lymphocytes and B cell infiltrates in lung tumors, but has limited prognostic value. Further studies will be required to evaluate the possible predictive role of PD-L1 expression in macrophages and other immune cell types.

